# The N-terminal acetyltransferase Naa10 is essential for zebrafish development

**DOI:** 10.1042/BSR20150168

**Published:** 2015-09-18

**Authors:** Rasmus Ree, Line M. Myklebust, Puja Thiel, Håvard Foyn, Kari E. Fladmark, Thomas Arnesen

**Affiliations:** * Department of Molecular Biology, University of Bergen, N-5020 Bergen, Norway; † Department of Surgery, Haukeland University Hospital, N-5021 Bergen, Norway

**Keywords:** abnormal development, *Danio rerio*, Nα-acetyltransferase 10 (Naa10), N-terminal acetyltransferase A (NatA), N-terminal acetyltransferase (NAT), N-terminal acetylation

## Abstract

N-terminal acetylation, catalysed by N-terminal acetyltransferases (NATs), is among the most common protein modifications in eukaryotes and involves the transfer of an acetyl group from acetyl-CoA to the α-amino group of the first amino acid. Functions of N-terminal acetylation include protein degradation and sub-cellular targeting. Recent findings in humans indicate that a dysfunctional Nα-acetyltransferase (Naa) 10, the catalytic subunit of NatA, the major NAT, is associated with lethality during infancy. In the present study, we identified the *Danio rerio* orthologue zebrafish Naa 10 (zNaa10). *In vitro* N-terminal acetylation assays revealed that zNaa10 has NAT activity with substrate specificity highly similar to that of human Naa10. Spatiotemporal expression pattern was determined by *in situ* hybridization, showing ubiquitous expression with especially strong staining in brain and eye. By morpholino-mediated knockdown, we demonstrated that *naa10* morphants displayed increased lethality, growth retardation and developmental abnormalities like bent axis, abnormal eyes and bent tails. In conclusion, we identified the zebrafish Naa10 orthologue and revealed that it is essential for normal development and viability of zebrafish.

## Abstract (precis)

The Naa10 (Nα acetyltransferase 10) N-terminal acetyltransferase is implicated in cancer and developmental syndromes in humans. We show that its enzymatic activity is conserved in zebrafish, and that Naa10 depletion leads to developmental abnormalities.

## INTRODUCTION

Nα-terminal acetylation (Nt-acetylation), the process in which an acetyl group from acetyl-CoA is transferred on to the α amino group of a protein's first amino acid residue, occurs on the majority of eukaryotic proteins [1–4]. Though ubiquitous, its function has been the subject of some controversy. Whereas earlier work suggested that Nt-acetylation protects proteins from degradation [5,6], possibly by blocking ubiquitination of the N-terminus [7], more recent studies demonstrate that this modification is functionally implicated in targeting proteins for degradation via a novel branch of the N-end rule pathway, probably as a way of degrading misfolded proteins or maintaining appropriate stoichiometries of protein subunits [8–10]. Other direct roles for Nt-acetylation include modulating protein complex formation [11], preventing post-translational endoplasmic reticulum (ER)-translocation [12], as well as regulating protein function and subcellular localization [13–15].

In eukaryotes, there are up to six N-terminal acetyltransferases (NATs), termed NatA–NatF, serving as the catalytic machinery for Nt-acetylation. Each NAT is composed of one or more specific subunits and is active towards specific subsets of proteins, mostly determined by the N-terminal amino acid sequence of the substrate [16]. In terms of the number of substrates, the major NAT is NatA, which co-translationally acetylates N-termini starting with a small amino acid (Ala, Cys, Ser, Thr, Val, Cys), after the initiator methionine has been removed. Its substrate pool thus consists of almost 40% of all N-termini in humans [1]. The NatA complex consists of the catalytic subunit Nα-acetyltransferase (Naa)10 (Ard1, arrest defective 1) and the auxiliary subunit Naa15 (NATH, N-acetyltransferase human/Nat1) [17–19]. Naa50 (San, separation anxiety) is also found associated with the complex in yeast [20], *Drosophila* [21] and humans [22], as is the huntingtin yeast two-hybrid protein K in humans [23]. Free, non-complexed Naa10 was previously found to post-translationally Nt-acetylate the acidic β- and γ-actins [24]. Naa10 also possesses Nt-propionyltransferase activity [25], lysine acetyltransferase activity [26–28] and was shown to act as a binding partner to other proteins, modulating their activity in an acetyltransferase-independent manner [29,30]. Components of the NatA complex, in particular Naa10, have been linked to carcinogenesis, in both tumour promoter and tumour suppressor capacities [31]. *Saccharomyces cerevisiae naa10Δ* and *naa15Δ* strains show identical phenotypes with mating defects and temperature sensitivity, though they remain viable [19]. Loss of Naa10 function is lethal, however, in *Drosophila* [32], *Trypanosoma brucei* [33] and *Caenorhabditis elegans* [34]. The recently identified Ogden syndrome, where boys harbouring a mutation in *NAA10* die during infancy, strongly implies that defective Naa10 and impaired Nt-acetylation is lethal for humans [35,36]. A splice donor mutation in the *NAA10* gene was found to cause Lenz microphthalmia syndrome through dysregulation of the retinoic acid signalling pathway [37].

The number of affected substrates, the severity of knockdown phenotypes across several model systems and implications in disease and development all point to a critical role of Naa10 in normal cell function. In the present study, the zebrafish *Danio rerio* was used to investigate whether Naa10 is necessary for viability and early development in a vertebrate. We identified a zebrafish Naa10 (zNaa10) candidate and confirmed a high degree of similarity between its sequence and those of characterized Naa10-proteins from other species. The zNaa10-candidate was recombinantly expressed and purified and the substrate specificity, as assessed by *in vitro* Nt-acetylation assays, revealed that this protein indeed was zNaa10. The spatiotemporal expression pattern of the *naa10* gene at early stages was determined by whole-mount *in situ* hybridization. Finally, we knocked down *naa10 in vivo* to demonstrate that it has a critical role in early zebrafish development.

## EXPERIMENTAL

### Protein BLAST and phylogeny

Candidate proteins were found by entering the amino acid sequences of the human Naa10, Naa11, Naa20, Naa30, Naa40, Naa50 and Naa60 proteins into the BLASTp search engine (http://blast.ncbi.nlm.nih.gov/Blast.cgi, NCBI). Top ranked candidates in zebrafish were chosen based on the lowest E-value. A phylogenetic tree with these candidates, as well as the human Naa10–Naa60 and yeast Naa10 (ARD1), Naa20 (NAT3), Naa30 maintenance of the wild-type killer virus 3 (MAK3), Naa40 (NAT4) and Naa50 (NAT5) was constructed in Clustal Omega, using the neighbour joining method.

### Cloning of zebrafish *naa10*

Total RNA was isolated from 5 days post-fertilization (dpf) zebrafish larvae as described by Peterson and Freeman [38]. cDNA was made using the Transcriptor Reverse Transcriptase kit (Roche). *naa10*-specific primers (*naa10*- NcoI-F 5′-CTCCATGGCATGAATATACGCAACGCACG-3′ and *naa10*- Acc65I-R 5′-CTGGTACCTTAAGAGTCTGACGATGAGTC-3′) were used to amplify the *naa10* gene from the cDNA library. The PCR product was ligated into a pJET cloning vector [part of the CloneJet PCR cloning kit (Fermentas), using the Quick Ligation™ Kit (New England Biolabs)]. It was later subcloned into the pETM41 expression vector (G. Stier, EMBL). The pETM41 vector encodes a maltose-binding protein (MBP) N-terminally to the insert, as well as a C-terminal His-tag. The construct was termed pETM41-*naa10* and verified by DNA sequencing.

### Expression and purification of MBP–zNaa10 and MBP–hNaa10

One Shot® BL21 Star™ Chemically Competent *Escherichia coli* cells were transformed with pETM41–*naa10* or pETM41–*NAA10* encoding the MBP–hNaa10 (human Naa10) protein [24] and a 200 ml of culture was grown to a *A*_600_ of 0.6 (at 37°C), followed by transfer to 18°C and addition of IPTG to a final concentration of 1 mM. Cells were incubated at 18°C in a shaker at 250 rpm for 18 h and harvested the following day by centrifugation at 12100 ***g*** and 4°C for 15 min. Cell pellets were resuspended in 15 ml of lysis buffer [50 mM Tris/HCl, 0.3 M NaCl, 2 mM DTT, 1 tablet/50 ml of Complete EDTA free protease inhibitor cocktail (Roche), pH 7.4] and sonicated for 6 × 30 s. After sonication, the cell lysate was centrifuged once more for 15 min at 12100 ***g***. The supernatant containing the soluble protein fraction was added to a 5 ml HisTrap column (Amersham), washed with immobilised metal ion affinity chromatography (IMAC) wash buffer (50 mM Tris/HCl, 0.3 M NaCl, 2 mM DTT, 20 mM imidazole, pH 7.4) and eluted with IMAC elution buffer (same as wash buffer, but with 350 mM imidazole). Fractions containing recombinant protein were combined and subjected to gel filtration on a HiLoad 16/60 Superdex 75 column (Amersham) and eluted with gel filtration buffer (50 mM Tris/HCl, 0.3 M NaCl, 1 mM DTT, pH 7.4). Protein purity was confirmed by SDS/PAGE and Coomassie staining and concentration was checked by absorbance measurements at 280 nm.

### DTNB based Nt-acetylation assay

5-5′-dithiobis (2-nitrobenzoic acid) (DTNB) reacts with free thiol groups to give the product 2-nitro-5-benzoate (NTB^2−^), the concentration of which can be measured spectrophotometrically. Using the method of Thompson et al. [39], slightly modified [40], we quantified the formation of NTB^2−^ after reaction of DTNB with an acetyltransferase assay sample. The acetyltransferase assay was performed by incubating purified MBP–zNaa10 (120 nM) or MBP–hNaa10 (50 nM) in acetylation buffer (50 mM Tris/HCl, 1 mM DTT, 0.2 mM EDTA, pH 8.5) with 300 μM substrate peptide (Biogenes) and 300 μM acetyl-CoA (Sigma–Aldrich). Reactions were stopped with two times the volume of quenching buffer (3.2 M guanidinium/HCl, 100 mM sodium phosphate dibasic, pH 6.8) after 10 min at 37°C. To measure CoA production, DTNB (2 mM final, dissolved in 100 mM sodium phosphate dibasic, pH 6.8 and 10 mM EDTA) was added to the quenched reaction and the absorbance at 412 nm was measured. Thiophenolate production was quantified assuming *λ*=13.7 × 103 M^–1^ · cm^–1^. Background absorbances were determined and subtracted from the absorbance determined for each individual reaction. Activity towards EEEIA peptide substrate was defined as 100% enzyme activity and activity towards other peptide substrates was reported relative to this. Assays were performed in triplicate and turnover for the limiting substrate did not exceed 10%.

### Synthetic substrate peptides

Peptides were custom made (Biogenes), varying in their seven N-terminal residues [DDDIAAL (corresponding to the N-terminus of β-actin), EEEIAAL (γ-actin), SESSSKS (high-mobility group protein A1) and MLGPEGG (heterogenous nuclear ribonucleoprotein F) but had the same 17 C-terminal residues (RWGRPVGRRRRPVRVYP[OH]). The common C-terminal segment is identical with the adrenocorticotropic hormone, with lysines replaced by arginines to ensure that no Nε-acetylation interfered with the activity measurements.

### Housing, care and microinjection of zebrafish

Animals were housed at the zebrafish facility at the Department of Molecular Biology, University of Bergen. The facility is run in agreement with the European Convention for the Protection of Vertebrate Animals used for Experimental and Other Scientific Purposes. This project was in agreement with the Norwegian Animal Welfare Act and The Norwegian Regulation on Animal Experimentation. No ethics committee approval was sought since this is not required for experiments on live embryos which are terminated within 5 dpf. Adult, spotty zebrafish were kept in continually running, osmotically cleaned water at 28.5°C on a 14/10 h light/dark cycle. Adults were fed twice daily with *Artemia* larvae. Embryos were kept in an incubator in E3 medium at 28.5°C for the first 5 days of life. Dead embryos were removed and E3 medium was replaced each day. Eggs were injected at the 1-cell stage with 1-2 pmol of morpholinos (GeneTools). For knockdown, a *naa10* morpholino oligonucleotide (MO) targeting the splice-site between intron 2 and exon 3 of the *naa10* gene was used (sequence: 5′-GGACAGCTTTAACAAAATGAACACA-3′). The control morpholino (Ctrl MO) used is a standard control (sequence: 5′-CCTCTTACCTCAGTTACAATTTATA-3′). The translation-blocking MO against *naa10* was 5′-GTGCGTT-GCGTATATTCATGTTGAC-3′, whereas that of the translation-blocking MO against p53 was 5′-GCGCCATTGCTTTGCAA-GAATTG-3′ and is previously validated [41].

### PCR validation of *naa10* splice inhibition

RNA was isolated from 1 dpf embryos and used to make a cDNA library via reverse transcriptase, as described above. The aforementioned *naa10* primers were used to amplify *naa10* from the cDNA template. Diminished levels of *naa10* transcript (629 bps) were confirmed by agarose gel electrophoresis. DNA normalization/control PCR was done using the same cDNA and primers designed for a segment of zebrafish β-actin (z-actb1-F-: 5′-TGTTGCCACCTTAAATGGCCT-3′ and z-actb1-R: 5′-TGTACAGAGACACCCTGGCT-3′), yielding a 454 bp product.

### *In situ* hybridization

*naa10* was amplified from cDNA using *naa10*-specific primers, as mentioned above, and cloned into pCR®II TOPO vector (Invitrogen). The sequence and orientation of the insert was confirmed by sequencing. Anti-sense RNA probe was synthesized using Sp6 polymerase (New England Biolabs) and digoxigenin-labelled ribonucleotides (Roche). For the negative control, sense RNA was synthesized using T7 polymerase (New England Biolabs). Embryos were fixed in 4% paraformaldehyde (PFA) overnight at 4°C. After washing with 1× PBS, embryos were dehydrated with methanol and stored at −20°C. Rehydrated embryos were washed with PBSTw (PBS, 0.1% Tween, 0.2% BSA). Twenty-four hours post-fertilization (hpf) and older embryos were permeabilized with proteinase K (10 mg/ml) and post-fixed in 4% PFA. Embryos were pre-hybridized for 2 h in hybridization buffer (50% formamide, 5× saline-sodium citrate (SSC), 0.5 mg/ml yeast tRNA, 50 μg/ml heparin and 0.1% Tween-20) at 65°C and thereafter hybridized with 500 ng/ml RNA probe in hybridization buffer overnight. Washes were carried out at 65°C in 50% formamide in 2× SSCTw (2× SSC, 0.1% Tween-20), 2× SSCTw, thereafter 0.2× SSCTw, followed by 1× maleic acid buffer, pH 7.4. Embryos were blocked at room temperature in 1× maleic acid buffer, pH 7.4, 1% blocking agent (Roche) prior to overnight incubation with alkaline phosphatase conjugated anti-digoxigenin antibody (1:2500) at 4°C and then incubation with BM Purple substrate (Roche). Embryos were cleared using Murray's clear and imaged using a Leica m420 microscope with a CoolSNAP-Pro digital camera (Media cybernetics).

## RESULTS

### The putative zebrafish Naa10 is highly similar to Naa10 of other eukaryotes

The amino acid sequences of the known human NATs (Naa10, Naa11, Naa20, Naa30, Naa40, Naa50 and Naa60) were entered into the pBLAST search engine, searching the zebrafish proteome. We termed top-ranked results zNaa10–zNaa60. Each query gave an unambiguous top candidate (E-score > e-105). Human Naa10 and Naa11 gave the same top candidate, namely the putative zNaa10. These putative NAT sequences, along with the human NATs and the yeast NATs [yNaa10–yNaa50 (*Saccharomyces cerevisiae* Naa)] were aligned and a phylogenetic tree was constructed (Figure 1). This tree shows that the different zebrafish proteins cluster along with their predicted counterparts in yeast and human.

**Figure 1:**
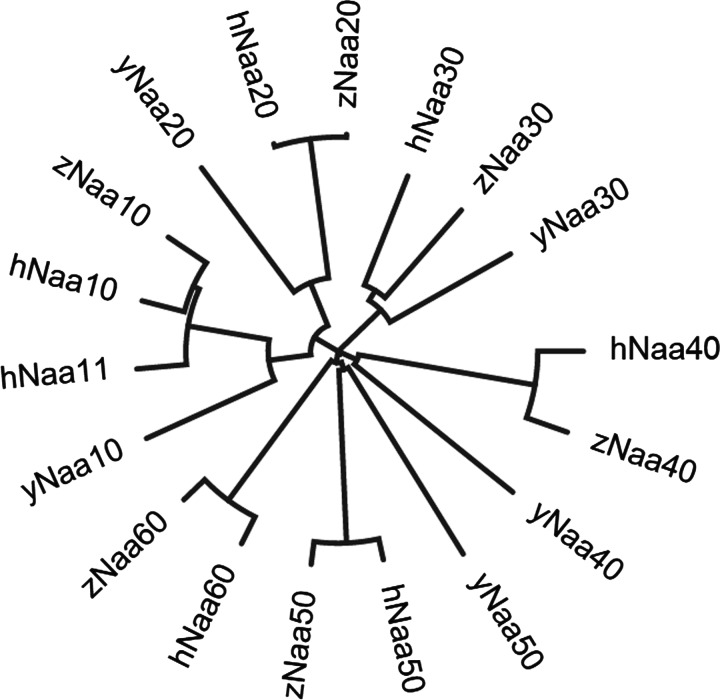
Phylogenetic tree showing the relationship between predicted zebrafish NATs and known NATs in humans and yeast Tree made in Clustal Omega using the neighbour joining method.

Using the same approach, top-ranked results for hNaa10 from *Pan troglodytes, Mus musculus, Rattus norvegicus, Danio rerio, Drosophila melanogaster, C. elegans, Arabidopsis thaliana* and *S. cerevisiae* were aligned and residues known to be important for substrate binding were identified (Figure 2). The sequence alignment confirms that the putative zebrafish Naa10 is highly similar to human Naa10. The acetyl-CoA-binding domain (RxxGxG/A) is entirely conserved, and the amino acid residues known to be involved in peptide substrate binding [42] are conserved as well. These key residues are labelled by * and include Leu22, Glu24, Tyr26, Arg113 and Tyr139. The conservation of these key residues indicates that Naa10 function is conserved among eukaryotes.

**Figure 2:**
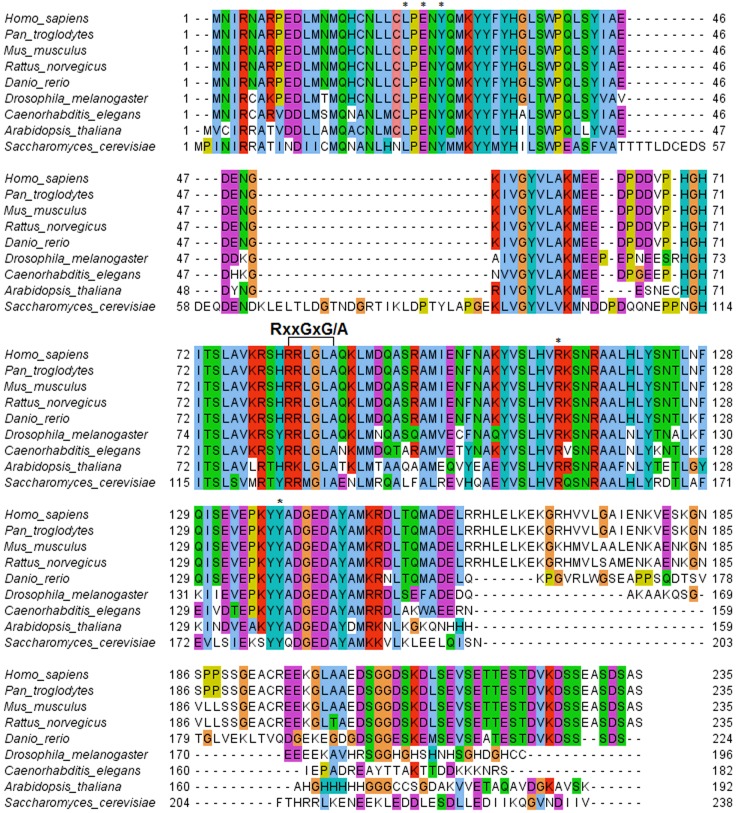
Alignment of the Naa10 protein sequences from several organisms The RxxGxG/A acetyl-CoA-binding domain is highlighted, as are Leu22, Glu24, Tyr26, Arg113 and Tyr139 (*) contributing to substrate peptide binding. Legend: light blue: non-polar amino acid; green: polar amino acid; purple: acidic amino acid; red: basic amino acid; turquoise: tyrosine and histidine; yellow: proline; orange: glycine.

### Recombinant purified zNaa10 Nt-acetylates typical Naa10-substrates

In order to show biochemically that zNaa10 indeed is a true Naa10 orthologue, we assessed its catalytic activity. zNaa10 and hNaa10 were purified as MBP-conjugates and tested for NAT-activity against known Naa10 substrates, as well as substrates known to be acetylated by Naa50 and by the NatA complex. The purified hNaa10 protein has an *in vitro* catalytic preference for acidic N-termini (aspartic acid and glutamic acid), preferring glutamic acid over aspartic acid, whereas serine-peptides (SESS), representing classical NatA complex substrates, are acetylated to a lower degree [24]. Purified MBP–zNaa10 displays the same order of preference as earlier shown for hNaa10 (Figure 3): EEEI is acetylated 3-fold more than the DDDI substrate, which again is preferred almost 2-fold to SESS, the canonical NatA substrate. MLGP, a non-NatA/Naa10 substrate *in vivo*, is only poorly acetylated. This verifies that zNaa10 is a NAT of the Naa10-type and that it has identical substrate specificity to hNaa10 *in vitro*.

**Figure 3:**
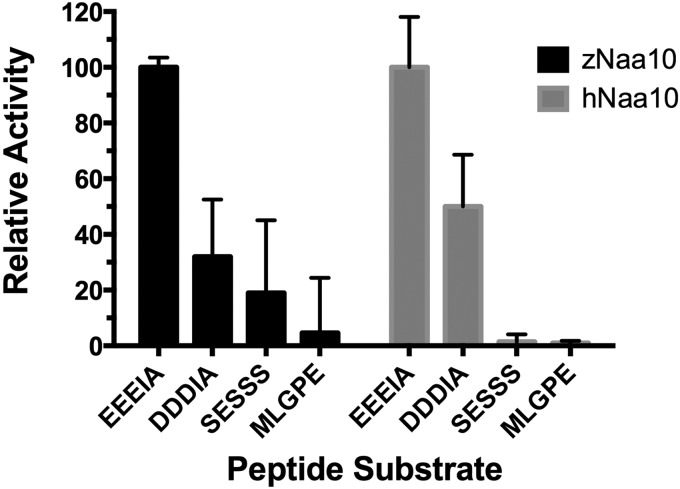
*In vitro* NAT activity of MBP–zNaa10 and MBP–hNaa10 towards peptide substrates The indicated synthetic oligopeptides were incubated with purified MBP–zNaa10 or MBP–hNaa10 enzyme in acetylation buffer at 37°C for 10 min. Product formation using EEEIA (Naa10 substrate) as substrate peptide was defined as 100% enzyme activity for each enzyme. The product formation using DDDIA (Naa10 substrate), SESSS (NatA substrate) and MLGPE (Naa50/NatE substrate) as substrate peptide was correlated to the product formation using EEEIA as substrate peptide. The assays were performed in triplicate (blanks were in duplicate) with error bars indicating S.D.

### *naa10* is expressed ubiquitously in early embryonic stages

We determined the expression pattern of *naa10* in early zebrafish development by *in situ* hybridization (Figure 4). *naa10* mRNA could be detected at all stages of development from the one-cell stage onwards (Figure 4A). The expression was found to be ubiquitous in early embryonic stages. At 24 hpf, the expression was found in forebrain, midbrain, hindbrain, retina, otic vescicles, myotomes and pronephric duct, though stronger expression was observed in the midbrain, retina and the pectoral fins (Figure 4). At 3 dpf, pronephric duct, pectoral fins, branchial arches and the midbrain, as well as the midbrain–hindbrain boundary, showed a stronger expression. To ensure that the staining was specific, we used the sense strand of the gene as a negative control (Figure 4F).

**Figure 4:**
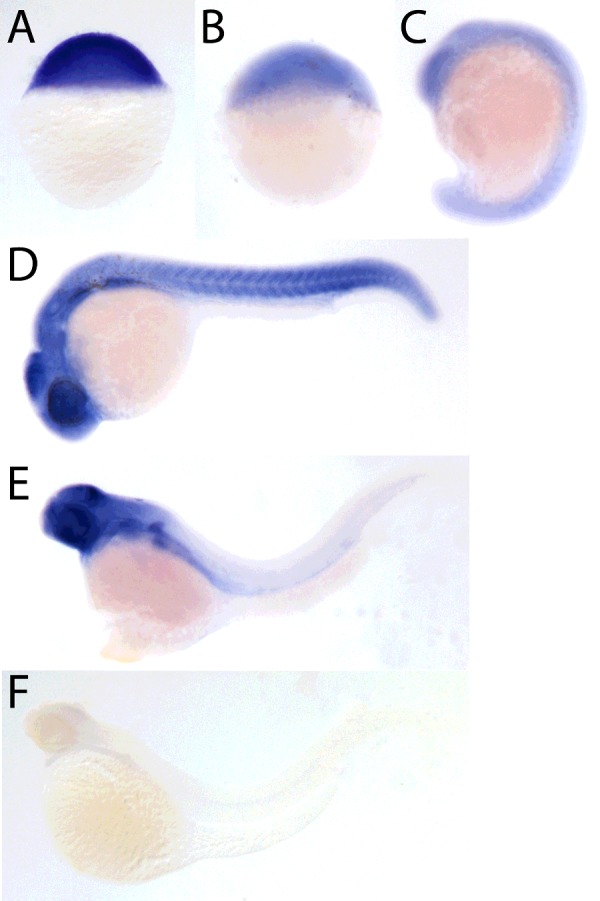
Spatiotemporal expression of naa10 revealed by *in situ* hybridization Lateral view of stained embryos at 0 hpf (**A**), 4 hpf (**B**), 18 hpf (**C**), 24 hpf (**D**) and 72 hpf (**E**). Negative control using the sense probe at 72 hpf (**F**).

### Morpholino-based knockdown of zNaa10 induces lethality and abnormal development

To determine whether Naa10 is essential for zebrafish viability and development, we targeted embryos with *naa10*-specific splice blocking MO [1]. Initially we determined a suitable dose of *naa10* MO for knockdown studies, yielding a clear phenotype but without significant off-target effects or mortality. A dose of 2 pmol yielded significant differences in survival between controls and morphants (Student's t-test, *P*<0.05) at 24 hpf. Since MOs may have unspecific, p53-mediated cell death as a side effect [43], we also performed co-injections with a p53 MO [41] to see if this was the case with our splice blocking MO. Co-injection of a p53 MO neither changed the survival rate nor the incidence of phenotypes (Supplementary Figure S1). Injection of an identical dose of a *naa10* translation- blocking morpholino yielded similar phenotypes as the Naa10-splice blocking MO (Supplementary Figure S2). We performed reverse transcriptase-PCR (RT-PCR) of 1 dpf embryos treated with 1 pmol *naa10* MO (Figure 5A). We concluded that levels of *naa10* transcript were significantly decreased in morphants. Figure 5B shows representative morphant and control embryos clearly demonstrating eye abnormalities and reduced pigmentation. Body length measurements revealed that morphants were significantly shorter than controls (Figure 5C). We also recorded the incidence of morphological abnormalities, namely bent axes, malformed or missing eyes or missing tails, at 2 dpf and found a markedly higher incidence in morphants compared with controls (Figure 5D). Five dpf embryos are also short and show morphological abnormalities (Figure 6). Embryos are bent (Figure 6C), have underdeveloped mandibles and have cardiac oedema and enlarged yolk sacs (Figure 6D).

**Figure 5:**
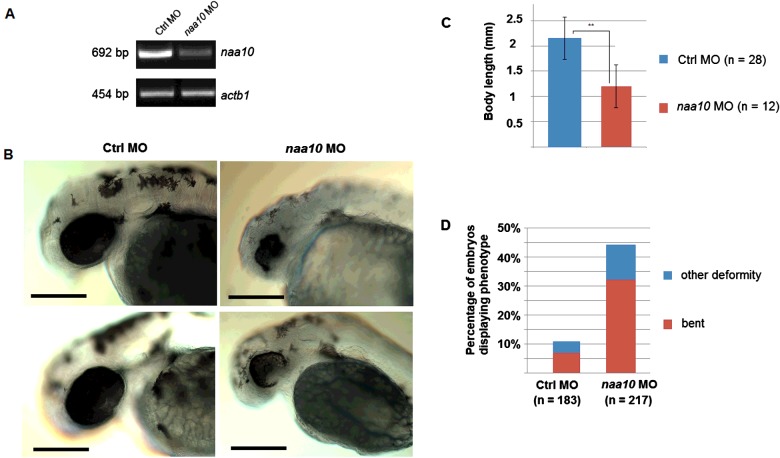
Knockdown of *naa10* leads to mortality and deformities (**A**) Knockdown in injected embryos verified by RT-PCR using zNaa10-specific primers. Embryos from two independent experiments were injected with 1 pmol MO and RNA was isolated at 1 dpf. β-Actin is used as control. (**B**): Two dpf embryos show impaired growth, eye abnormalities and reduced pigmentation. (**C**): Body length of control and *naa10* morphants at 2 dpf. **P<0.01 (Student's *t* test). (**D**): Percentage of injected embryos with morphological abnormalities at 2 dpf. Highlighted are percentages of bent embryos (red). Other abnormalities included loss of eyes or deformed eyes or other gross morphological defects. Scale bar=0.2 mm.

**Figure 6:**
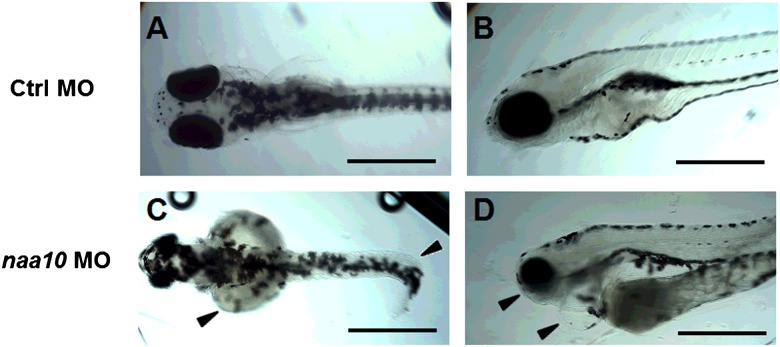
Morphological defects in *naa10* morphants at 5 dpf At 5 dpf, *naa10* morphants had a reduced head size, underdeveloped jaws and eyes, enlarged yolk sacs, pericardial oedema and curled tails (arrows in **C** and **D**) when compared with controls (**A** and **B**). At this stage *naa10* morphants exhibited normal pigmentation. Figure shows 5 dpf embryos injected with 1 pmol of Ctrl MO or *naa10* MO. Scale bar=0.5 mm.

## DISCUSSION

The comparison of known and putative Naa10 proteins from several organisms highlights the evolutionary conservation from budding yeast to mammals (Figure 2). Using pBLAST, we could find no evidence of other proteins that would be probable candidates for the zebrafish *naa10* orthologue (E-score: 1e-126). Predicted NAT orthologues to the other human NATs (zNaa20–zNaa60) ranked well below zNaa10 (E-scores between e-3 and e-19). Our database searches did not give any evidence of *naa10* splice variants. zNaa10 also shows high sequence similarity to the human paralogue, hNaa11. Naa11 is a mammal-specific Naa10 paralogue that originated from a retrotransposition event [44]. It has a high sequence similarity to the other Naa10-type proteins (Figure 1), but we could find no evidence of a similar paralogue in zebrafish by searching the zebrafish proteome.

Apart from two stretches near the N-terminus in yeast, which suggest an insertion event (as the same sequence is neither seen in metazoans, nor in *A. thaliana*), most of the zNaa10 protein is highly conserved among all eukaryotic species investigated (Figure 2). Of particular interest is the conserved acetyl-CoA-binding domain RxxGxG/A (highlighted in Figure 2), as well as Leu22, Glu24, Tyr26, Arg113 and Tyr139 (*). The latter five amino acid residues contribute to substrate binding and catalysis as demonstrated by the solved structure of the NatA complex from *Schizosaccharomyces pombe* [42]. The conservation of these critical motifs strongly supports zNaa10 as a candidate for the zebrafish Naa10 homologue.

The *in vitro* acetylation assays verified that zNaa10 is a NAT of the Naa10-type and that it has identical substrate specificity to human Naa10 (Figure 3). The specificity of this non-complexed Naa10 towards the acidic N-termini is thought to represent a post-translational NAT-activity acetylating the actins. Based on proteomics data in human cells, γ- and β-actin are Nt-acetylated and are likely targets of Naa10 [24]. Some actins have acidic N-termini in both humans and zebrafish. The strong preference of zNaa10 for acidic N-termini *in vitro* suggests that it is capable of acetylating endogenous zebrafish β- and γ-actin N-termini.

Whole-mount *in situ* hybridization was used to visualize the expression pattern of *naa10* at various developmental stages. *naa10* is found to be maternally expressed and is found throughout the developing embryo (Figure 4). Especially strong staining can be seen in eyes, myotome, fin buds and the dorsal midbrain. As the Naa10 protein is thought to be an essential component of the protein processing machinery in all cells, the ubiquitous expression was expected. Given that knockdown affects eye development (Figure 5B), it is also interesting to see that expression in the eye is high. Recently, a study of patients with Lenz microphthalmia concluded that loss of retinoic acid signalling plays a role in the development of this disorder [37]. The small, underdeveloped eyes seen in *naa10* morphants fits with these clinical findings.

The morpholino experiments suggested that a complete loss of Naa10 may cause lethality. Lowering the dose to 1 pmol gave no significant difference in mortality between treated groups, as well as an acceptable level of overall mortality and a clear incidence of phenotypes. Thus, this dose was chosen in further experiments. Because of the lack of a suitable antibody against zNaa10, we were not able to validate the translation blocking MO at the protein level. However, RT-PCR analysis of embryos treated with the splice blocking MO shows a significant decrease in *naa10* mRNA levels (Figure 5B). We did not observe any band corresponding to mis- or un-spliced *naa10* transcripts, but the low level of normal *naa10* transcript seen in Figure 5B indicates that the splice blocking MO has been effective and that mis-spliced transcript may have been degraded.

Loss of zNaa10 was associated with adverse effects on growth and development. At 2 dpf, morphants were significantly shorter than controls (Figure 5C) and had higher incidence of abnormalities, such as bent spines or tails. In addition, eyes were small and underdeveloped, and embryos showed less pigmentation. At the later stage (5 dpf), morphants had normal pigmentation, but were still smaller than controls and enlarged yolk sacs indicated decreased growth and activity (Figure 6). Morphants at 5 dpf also showed underdeveloped mandibles and pericardial oedema (Figure 6D). How Naa10 exerts its effects on a developmental level is not known. It is presumed to act on a large number of substrates involved in a multitude of different cellular processes. It was earlier established that Ogden syndrome, a rare genetic disorder, is caused by a mutation in *NAA10* which severely inhibits the catalytic activity of the mutant protein. One of these studies found that cells expressing the mutant protein had reduced cell proliferation, mediated by perturbation of the Retinoblastoma 1 pathway [35,36].

Importantly, these syndromes are caused by mutations which render the protein dysfunctional, but still retaining some catalytic activity. The findings of the current study are in good agreement with the developmental importance of *NAA10* inferred from the clinical cases.

The phenotype seen in *naa10* morphants has some overlap with a more generalized, ‘monster’ phenotype, described as an unspecific side effect of MO treatment [45]. Unspecific cell death has also been linked to sequence-independent morpholino induction of a p53 response [43]. We believe that the similar deformities seen with both splice- and translation-blocking morpholinos against *naa10*, as well as the lack of effect of a p53 MO, indicates that these phenotypes are indeed specific. As Naa10, due to its numerous substrates, is probably implicated in a multitude of cellular processes, a generalized, multi-system knockdown phenotype is to be expected. Such a prediction is also supported by the ubiquitous expression profile as revealed by *in situ* hybridization. However, the exact mechanism by which *naa10* knockdown leads to this generalized developmental delay is not known. Because Naa10/NatA has so many substrates, it is likely that *naa10* morphants are affected in a number of molecular pathways. This may create a plethora of distinct and overlapping phenotypes in several tissues. The fact that *naa10* is ubiquitously expressed at all surveyed stages of development supports this interpretation of the phenotype.

It seems reasonable to think that a loss of actin acetylation may be of some importance in the morphological abnormalities seen in *naa10* morphants. Actin is a central component of the cytoskeleton and is involved in cell motility during normal development [46,47]. In yeast, the charge of the N-terminus is functionally important in tropomyosin binding [48]. For yeast actin, a loss of acetylation has been shown to decrease the affinity of its N-terminal for myosin binding [49].

Naa10, the catalytic subunit of the major eukaryotic NAT, is conserved among all surveyed model organisms from yeast to humans, now including zebrafish. zNaa10 has the same substrate specificity as hNaa10. This suggests that zNaa10 may have the same molecular roles as the human Naa10 protein and that zebrafish is a suitable model for studying the organismal and developmental importance of this protein. Our results indicate that Naa10 is essential for viability and normal development in zebrafish, with knockdown resulting in severe and reproducible phenotypes, including generalized malformations and short body length. Given the large number of likely Naa10 substrates, large, multi-systemic effects were expected and were also observed. Further studies should elaborate on the distinct pathways and molecular targets affected by *naa10* MO.

## Abbreviations

Ard1: arrest defective 1
Ctrl MO: control morpholino oligonucleotide
dpf: days post-fertilization
DTNB: 5-5'-dithiobis(2-nitrobenzoic acid)
hNaa: human Naa
hpf: hours post-fertilization
IMAC: immobilised metal affinity chromatography
MBP: maltose-binding protein
MO: morpholino oligonucleotide
Naa: Nα acetyltransferase; NAT, N-terminal acetyltransferase; Nt, Nα-terminal; PFA, paraformaldehyde; RT-PCR, reverse transcriptase PCR; SSC, saline-sodium citrate; SSCTw, saline-sodium citrate with Tween-20; yNaa, Saccharomyces cerevisiae Naa; z-actb1, zebrafish β-actin; zNaa, zebrafish Naa
